# Promoter Effect of Pt on Zr Catalysts to Increase the Conversion of Furfural to γ-Valerolactone Using Batch and Continuous Flow Reactors: Influence of the Way of the Incorporation of the Pt Sites

**DOI:** 10.1021/acs.energyfuels.4c01174

**Published:** 2024-05-24

**Authors:** Adrian García, Anna Saotta, Pablo J. Miguel, Rita Sánchez-Tovar, Giuseppe Fornasari, Alessandro Allegri, Benjamín Torres-Olea, Juan Antonio Cecilia, Stefania Albonetti, Nikolaos Dimitratos, Benjamin Solsona

**Affiliations:** † Department of Chemical Engineering, Universitat de València. Av. Universitat s/n, Burjassot, 46100 Valencia, Spain; ‡ Department of Industrial Chemistry “Toso Montanari”, Università di Bologna, Viale Risorgimento 4, Bologna 40136, Italy; § Department of Inorganic Chemistry, Crystallography and Mineralogy, Campus de Ciencias, Universidad de Málaga, 29071 Málaga, Spain

## Abstract

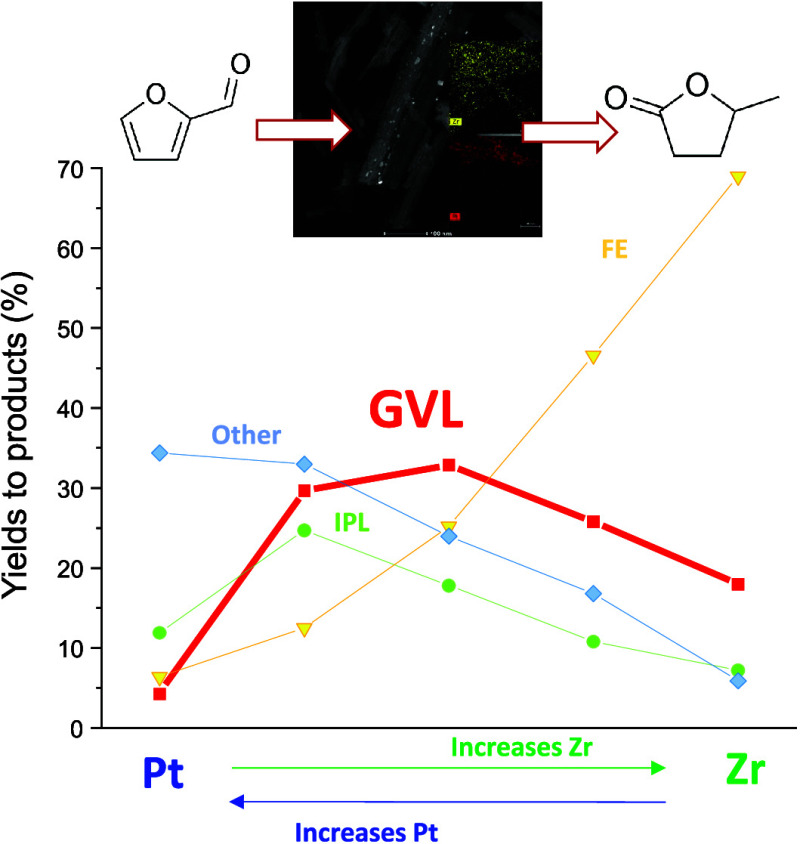

The valorization of biomass and its transformation into fuels are highly interesting due to the abundance of biomass and its almost neutral carbon emissions. In this article, we show the production of γ-valerolactone (GVL), a valuable product, from furfural (FF), a compound that can be easily obtained from biomass. This FF to GVL transformation involves a catalytic cascade reaction with two hydrogenation steps. Pt and/or Zr supported on sepiolite catalysts have been prepared and tested in the FF transformation reaction. A physical mixture of a Zr-based and a Pt-based catalyst has reached a yield to GVL of ca. 50% after 16 h at 180 °C. This performance largely exceeds that obtained by each of the single Pt or single Zr metal catalysts independently, showing a strong synergistic effect. These data suggest that each metal (Pt and Zr) plays an important and complementary role in different reaction steps. Furthermore, the physical mixture appears to be much more efficient than bimetallic Pt/Zr catalysts synthesized with the same amount of metals. The role of the type of acidity and the oxidation state of the surface platinum species on the catalytic performance has been discussed. Moreover, this reaction has been carried out in batch and continuous flow reactors, and a comparative study between the two operation modes has been undertaken. A certain correlation between the catalytic results obtained by both operation modes has been found.

## 1 Introduction

The amount of energy consumption in society has increased in recent decades. Unfortunately, the biggest share of energy production belongs to nonrenewable energy sources.^1,2^ Additionally, the excessive consumption of fossil fuels has caused several environmental and health problems due to pollution and has increased the greenhouse effect due to the CO_2_ release.^3^ For these reasons, new alternatives to produce sustainable energy, such as the use of biomass, are being currently studied. Biomass is abundant and has an almost neutral carbon balance because the amount of CO_2_ released in the combustion process is equal to the amount of CO_2_ captured by trees and plants through the photosynthesis process.^1,4,5^ It is important to note that biomass can be treated by enzymatic or thermochemical processes to isolate and valorize its components.^6−11^ One of the most desired components is lignocellulosic biomass since it is nonedible, abundant, and cheap. Many different catalytic routes have been developed to valorize the lignocellulosic biomass into valuable chemical compounds and energy production.^12−14^

One of the main products obtained from the sugar platform is furfural (FF). This compound is easily obtained through the hydrolysis of the hemicellulose fraction using acids to form pentoses and the subsequent dehydration to produce FF.^15,16^ FF can be used to produce a wide range of different valuable chemical products used in oil refining, plastics, and agrochemical and pharmaceutical industries, among others.^15,17^ One of these compounds is γ-valerolactone (GVL), which is a green and nontoxic molecule with many applications.^18^ Thus, apart from the promising use of enzymes or ionic liquids, GVL can be employed as a solvent to pretreat the lignocellulosic biomass.^19,20^ In any case, the main application of GVL is as a biofuel or fuel additive due to its high combustion energy, which is similar to that of ethanol.^21^ Moreover, GVL can also be used as a precursor of different compounds, such as 1,4-pentanediol, pentenoic acid, or butadiene.^22−24^

The production of GVL from FF involves two hydrogenation reactions, so a source of hydrogen is needed to form GVL.^25^ The typical hydrogen sources reported to carry out the reaction have been a range of different alcohols, such as 2-propanol, 2-butanol, or ethanol, due to the facile availability of H atoms, which promotes the reduction of aldehydes and/or ketones into their respective alcohols, while the secondary alcohol used as sacrificing alcohol is oxidized to ketones.^26,27^

The transformation of FF into GVL involves cascade reactions with different intermediates that require the mediation of a catalyst to direct the process toward the selective one-step production of GVL.^28^ The use of different metals supported in different types of materials has been used as heterogeneous catalysts to produce GVL from FF in one step.^29−32^ Among them, zirconia has been the most studied metal oxide due to the high yields of GVL obtained through consecutive reactions.^33−35^ Zr species, which provide Lewis acid sites (LAS), have been typically deposited or incorporated on zeolite frameworks. Zeolites are known to provide Brønsted acid sites (BAS). It has been reported that both acid sites, LAS and BAS, are essential in the different steps of the cascade reaction process.^25,36^

Noble metal-based materials have not been widely reported as catalysts for the synthesis of GVL from FF as the yields obtained are often very poor. However, there are different studies, where catalysts containing noble metals, such as platinum, can effectively transform FF to produce furfuryl alcohol (FAL) through C=O hydrogenation.^37−39^ Interestingly, several studies have demonstrated that the improvement of the catalytic activity and the selectivity to FAL is due to the enhanced dispersion of supported Pt nanoparticles.^40,41^ In this way, Bhogeswararao and Srinivas^42^ used Pt supported over γ-Al_2_O_3_, achieving a yield to FAL of 65.5% under a H_2_ pressure of 60 bar and 25 °C, while Wang et al.^43^ synthesized a Pt/MWNT catalyst, obtaining a yield toward FAL of 75% at 20 bar and 5 h of reaction.

There are studies in which Pt is used in bimetallic catalysts with a low loading of Pt (1 wt %), improving the catalytic activity of the oxide or zeolite in the transformation of FF.^37,40^ However, there are no studies using Pt to produce GVL from FF in a one-pot process. In this work, the addition of a small amount of Pt onto zirconium-containing catalysts was explored. Catalysts containing both Zr and Pt synthesized in different ways as well as physical mixtures of catalysts containing only Zr and Pt were tested in the FF transformation. As most of the works about this reaction have been carried out in batch and only a few have been undertaken in continuous regime,^44^ we have assessed the catalytic behavior of these catalysts using both types of reactors: batch and continuous flow reactors.

## 2 Material and Methods

### 2.1 Preparation of Catalysts

Raw sepiolite collected from Toledo (Spain) and provided by Sepiolsa was used as a support to synthesize catalysts with zirconium or platinum, which were incorporated by the incipient wetness impregnation method.

The Zr-based catalyst was synthesized by dissolving zirconium(IV) oxynitrate hydrate ZrO(NO_3_)_2_·*x*H_2_O (99% purity) from Sigma-Aldrich (St. Louis, MO) in distilled water, and later, sepiolite was added in order to obtain a catalyst with 10 wt % ZrO_2_. The mixture was placed on a hot plate stirrer, and it was stirred at 90 °C until the solvent was evaporated. Later, the catalyst was calcined at 400 °C for 3 h under static air in an oven. This catalyst was labeled as 10Zr/Sep.

The Pt-based catalyst was synthesized using platinum(II) acetylacetonate as a precursor, which was dissolved in acetone. After that, sepiolite was added in order to obtain 1 wt % Pt. The mixture was stirred at 50 °C until the solvent was evaporated. Later, the catalyst was calcined at 400 °C for 3 h under static air in an oven. The catalyst was labeled as 1Pt/Sep.

Moreover, Zr or Pt was added to the catalysts 1Pt/Sep or 10Zr/Sep, respectively, in order to form a bimetallic catalyst. On the one hand, zirconium(IV) oxynitrate hydrate was dissolved in distilled water, and later, the synthesized catalyst 1Pt/Sep was added. The mixture was stirred at 90 °C until the solvent was evaporated. Later, the catalyst was calcined at 400 °C for 3 h under static air in an oven. This catalyst was labeled as 10Zr/1Pt/Sep. On the other hand, platinum(II) acetylacetonate was dissolved in acetone, and later, 10Zr/Sep was added and the mixture was stirred at 50 °C until the solvent was evaporated. Finally, the catalyst was calcined at 400 °C for 3 h under static air in an oven. The catalyst was labeled as 1Pt/10Zr/Sep.

### 2.2 Characterization Techniques

Catalysts were thoroughly characterized using different techniques. To determine the surface area of the catalysts, N_2_ adsorption was undertaken at −196 °C and using a Micromeritics Tristar apparatus with an enhanced secondary void system after outgassing at 150 °C before reaching vacuum conditions. The BET method was used to determine the surface area.

Powder X-ray diffraction (XRD) patterns of catalysts were collected between 10 and 80 ° at 2θ using an Enraf Nonius FR590 sealed tube diffractometer (Bruker, Delft, The Netherlands) equipped with a monochromatic Cu Kα1 source (40 kV and 30 mA).

High-resolution transmission electron microscopy (HR-TEM) analysis was conducted using an FEI Talos F200X, combining TEM imaging, high-resolution STEM, and energy-dispersive X-ray spectroscopy (EDS) signal detection. The catalysts were dispersed in ethanol, and a drop of the suspension was put on a Formvar/carbon-supported Cu grid (300 mesh).

The total acidity of the samples was analyzed with the temperature-programmed desorption of ammonia (NH_3_-TPD). 100 mg of each sample was used for the analysis. First, they were pretreated under helium flow at 550 °C. After cooling the samples, the adsorption of ammonia was carried out at 100 °C. The NH_3_-TPD analysis was conducted by increasing the temperature from 100 to 500 °C with a heating rate of 10 °C/min, and it was maintained at 500 °C for 15 min in helium (40 mL/min). A TCD detector was employed to quantify the evolved ammonia.

In order to investigate the Lewis and Brønsted acidity, the adsorption–desorption of pyridine (Py) coupled with Fourier transform infrared spectroscopy (FTIR) was recorded in a Tensor 27 instrument (Bruker) with a Michelson interferometer. A He–Ne laser was used as an internal reference, and a DTGS infrared detector was also used. 64 accumulations were taken in transmission mode with a spectral resolution of 4 cm^–1^. The surface of the materials was cleaned by heating at 200 °C for 1 h and under a pressure of 0.1 mbar. Later, the samples were exposed to a pyridine atmosphere (200 mbar of pyridine vapor pressure) for 15 min at 50 °C. Physisorbed pyridine was eliminated by exposing the sample to a vacuum for 15 min after adsorption. The desorption was carried out at 100 and 200 °C, for 15 min each, under a vacuum.

The samples were analyzed by X-ray photoelectron spectroscopy (XPS) using a physical electronics PHI5700 spectrometer, with non-monochromatic Mg Kα radiation (300 W, 15 kV, and 1253.6 eV) and a multichannel detector. For recording the spectra, a constant pass energy mode at 29.35 eV, with a 720 μm diameter analysis area, was used. Acquisition and data analysis were accomplished with a PHI ACCESS ESCA-V6.0F software package, whereas charge referencing was measured against adventitious carbon (C 1s at 284.8 eV). To determine the binding energies, a Shirley-type background was subtracted from the signals, and the fitting of recorded spectra was carried out with the Gaussian–Lorentzian curve.

### 2.3 Batch Catalytic Tests

The transformation of FF into GVL in one pot was carried out in an autoclave reactor. The reactor has an internal part covered by a Teflon container of 25 mL, which fits with the steel walls. In a typical run, 0.25 mmol of FF was dissolved and mixed with 5 mL of 2-propanol. Later, 0.1 g of the catalyst was added. In the case of the reaction, where two different types of catalysts were used, 0.05 g of each catalyst was added to the reaction. After adding the catalyst, the reactor was purged with N_2_ for 1 min to remove oxygen, and later, it was sealed. The reactor was placed in a hot plate stirrer with a silicon bath at 180 °C and stirred at 500 rpm for the duration of the experiments. After the reaction was completed, the reactor was cooled in an ice bath for 15 min. The liquid products were collected by filtration for quantitative analysis.

### 2.4 Analysis of the Products in Batch

The products were analyzed by gas chromatography using a mod. 5890 GC instrument (Hewlett-Packard, Palo Alto, CA). Dodecane was used as an internal standard. The column used was an Agilent HP-1 column (30 m x 0.32 mm x 0.25 μm) coupled with a flame ionization detector (FID) at 240 °C and an injection port at 220 °C. The temperature program for the chromatographic cycle was as follows: (i) 35 °C isothermal for 30 min, (ii) a heating rate of 1.5 °C/min from 35 to 230 °C, and (iii) 230 °C isothermal for 30 min. Moreover, a gas chromatography–mass spectrometer was used (GC-MS5977A MSD-7890A, Agilent, Santa Clara, CA) to identify other reaction byproducts. The temperature program used was the same as that used in the GC-FID.

### 2.5 Continuous Catalytic Tests

Continuous flow reactions were carried out by using a homemade liquid-phase fixed-bed reactor (Figure S1). An HPLC pump (JASCO PU4080i) feeds the solution to the reactor (R1), which is placed in an oven (E2); at the exit of the reactor, there is a VCR filter (F2), followed by a backpressure regulator (BPR). Silicon carbide (SiC) as the desired diluent was loaded into the reactor together with 1 mL (between 0.42 and 0.52 g) of catalyst placed within the isothermal zone of the oven.

In continuous flow conditions, contact time (τ) represents the time during which the flow, hence the reagent solution, stays in contact with the catalyst. To calculate this, the volume of the catalyst (*V*) and the volumetric flow rate (*f*) used are considered. In this work, the time of contact is expressed in minutes as follows
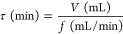


This parameter is different from the time on stream indicated in the graphs (see later) reported in the manuscript. In these cases, it represents the entire duration of the test that is being shown; hence, the time that the catalyst stays in contact with the reagent solution.

The diluent and the catalysts were sieved before (*d* > 60 and 80 < *d* < 60 mesh, respectively) to facilitate their separation at the end of the reaction. We want to mention that the catalysts tested were always in the form of 60/80 mesh (250/177 μm diameter) pellets. This size was chosen based on heuristics: catalysts used in continuous flow should generally be 1/10 in diameter compared to the inner diameter of the reactor. This rule should be applied while considering pressure drops, maximizing liquid and solid phase contact, and avoiding preferential paths through the catalyst. In our case, taking into account all of these parameters, we chose the size mentioned above.

Given that the hourly volumetric flow rate (6 mL/h) and the reaction volume (1 mL) used are the same in every test, the LHSV (liquid hourly space velocity) is always equal to 6 h^–1^.

The pressure and flow were stabilized in the reactor following the procedure already described in a previous study,^44^ which consists in pressurizing the reactor with nitrogen and then filling it with the substrate solution before initiating the heating process. Once the oven is at the desired temperature postreaction, liquid samples can be collected. A 67 mM furfural solution in 2-propanol, containing an equivalent of H_2_O and 330 μL of octane, used as the desired internal standard, was prepared in a 250 mL flask to be used as continuous feed. The samples were collected every hour at the end of the outlet of the reactor in a 10 mL volumetric flask and diluted with 2-propanol.

### 2.6 Analysis of the Products Obtained in the Continuous Flow Reactor

Postreaction samples were analyzed via gas chromatography (Shimadzu GC-2010 Pro) using a flame ionization detector (GC-FID). The analysis method used was as follows: the injector was heated to 280 °C for the vaporization of the mixture, with N_2_ flow as the eluent of 1.2 mL/min and a split ratio equal to 30; an Agilent HP-5 column (diameter 0.32 mm, length 30 m) was placed inside a heated chamber at a controlled temperature through the following temperature program: 2 min of isotherm at 50 °C at a heating rate of 10 °C/min up to 250 °C, followed by an isotherm of 2 min at 250 °C; and an FID detector was heated to 250 °C for compound detection. To calculate the response factors, the moles, hence the molar flow, and finally the conversion and selectivity of the different products obtained, calibration curves of the principal commercial molecules involved in the cascade reaction (furfural (FF), furfuryl alcohol (FAL), α- and β-Angelica lactone (α-AnL, β-AnL), FE (furfuryl propyl ether), γ-valerolactone (GVL), and isopropyl levulinate (IPL)) were constructed. Response factors and retention times were identified: 5.2, 5.5, 5.8, 7.0, 7.2, 7.3, and 9.9 min.

### 2.7 Calculations of Conversion, Yield, and Selectivity

According to eqs 1–4, furfural conversion, product selectivities, and the percentage of undesired products (others) were calculated, respectively
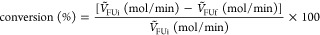
1
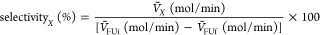
2
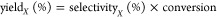
3
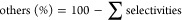
4where Ṽ_FUi_ and Ṽ_FUf_ are the initial and final molar flows of furfural (mol/min), while Ṽ_*X*_ is molar flow (mol/min) of X (where X identifies a certain product). All results are expressed as percentages.

### 2.8 Recycle of Catalysts Used in Batch Conditions

For the recycling test, we initially used 0.05 g of Pt/Sep +0.05 g of Zr/Sep at 180 °C for 8 h. We carried out this experiment three times in parallel with a fresh catalyst (first cycle). Then, we recovered the used catalysts by filtration and dried them at 180 °C for 12 h. The three recovered used mixtures were mixed, and then, we took two portions of 0.1 g and carried out in parallel two new experiments (second cycle). Then, after use, we recovered the mixtures by filtration, and after being dried, we used 0.1 g for the third cycle.

## 3 Results

### 3.1 Characterization Results

Table 1 presents the BET surface areas of the different catalysts synthesized. Sepiolite had a surface area of 226 m^2^/g. The deposition of metals caused a decrease in the surface area of the synthesized catalysts with respect to the support, especially for the catalyst 10Zr/1Pt/Sep, which presented 150 m^2^/g. The catalyst 1Pt/Sep presented a surface area of 180 m^2^/g, whereas the 10Zr/Sep and 1Pt/10Zr/Sep catalysts presented the highest surface area, very close to that of the support (sepiolite), with values of 200 m^2^/g.

**Table 1:** Surface Areas of the Support and the Catalysts

sample	*S*_BET_ (m^2^/g)	method	Pt/Zr (wt %)^b^
sepiolite^a^	226		
10Zr/Sep	200	Impregnation of Zr	0/9.7
1Pt/Sep	180	Impregnation of Pt	0.97/0
10Zr/1Pt/Sep	150	First Pt and then Zr	0.90/9.8
1Pt/10Zr/Sep	200	First Zr and then Pt	0.98/9.6

a Sepiolite calcined at 400 °C using the same cycle as the metal-containing catalysts.

b Chemical analysis of the bulk determined by ICP.

N_2_ adsorption/desorption isotherms of the catalysts used in this work are shown in Figure S2. In every case, a type IV isotherm with H3 hysteresis is observed. These results are characteristic of meso/macroporous materials like clays. Indeed, the adsorption increases significantly at *P*/*P*_0_ = 0.8–1, indicating the presence of pores with 10 < diameter <100 nm. However, a certain density of micropores is also present, given the visible initial adsorption. Pore distribution curves (Figure S3) confirm the latter interpretation, showing broad bands between 10 and 180 nm with a distribution maximum in the mesoporous range (23 < *d* < 45 nm) in all cases. These results are in line with the literature.^45^

**Figure 1:**
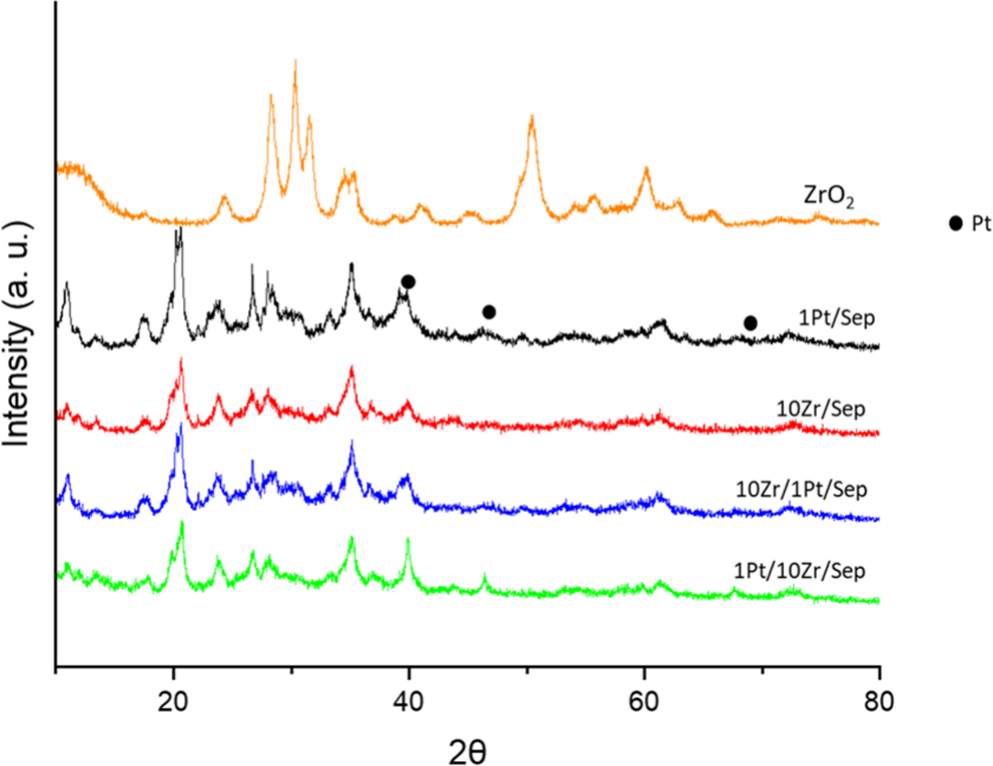
X-ray diffraction patterns of monometallic and bimetallic catalysts and pure ZrO_2_.

Figure 1 shows the X-ray diffraction patterns of the catalysts containing Pt and/or Zr and pure ZrO_2_. In the case of pure bulk ZrO_2_ catalyst, diffraction peaks at 2θ (°) = 17.5, 24.1, 28.1, 30.2, 31.6, 34.2, 34.6, 40.6, 50.3, 51.0, 60.0 were observed.^25,46^ These diffraction peaks correspond to ZrO_2_ in its monoclinic form (JCPDS: 37-1484). In addition, some reflections of ZrO_2_ in its tetragonal form (JCPDS 88-1007) at 2θ (°) = 30.2, 34.5, and 51.0° were also observed.^25^ No diffraction peaks related to ZrO_2_ were observed in the catalysts, which contained Zr. This could be due to the high dispersion of ZrO_*x*_ species on the surface of sepiolite or the presence of ZrO_2_ crystallites with a small crystallite size below 3 nm. Consistent with the low content of platinum, no diffraction peaks corresponding to phases containing platinum have been clearly observed although the presence of metallic Pt (JCPDS: 04-0802) cannot be completely discarded.^47−49^

**Figure 2:**
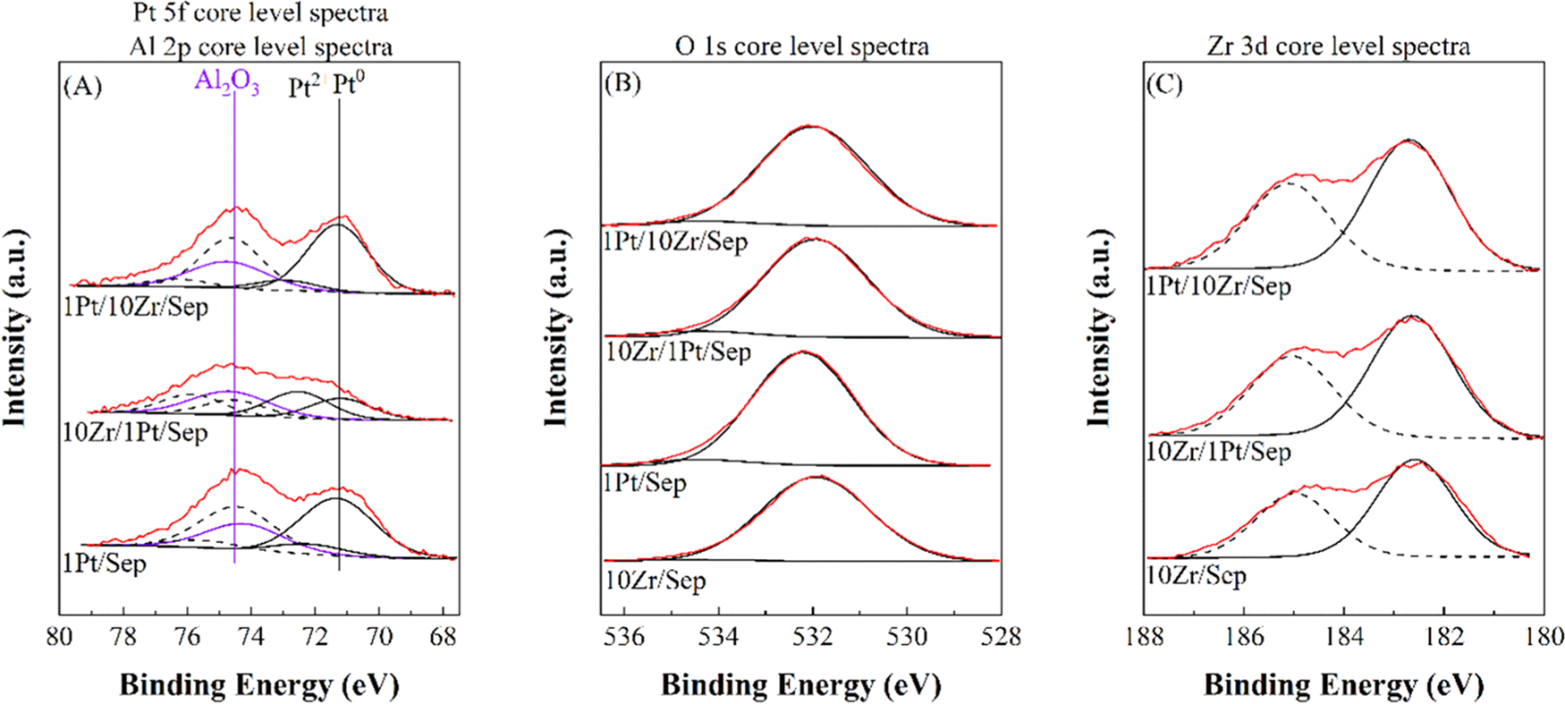
X-ray photoelectron spectroscopy (XPS) of monometallic and bimetallic catalysts: (A) Pt 5f and Al 2p core level spectra, (B) O 1s core level spectra, and (C) Zr 3d core level spectra.

The near surfaces of these catalysts were studied by XPS (Figure 2). In Table 2, the results of the XPS analysis show the near-surface chemical composition of the catalysts, as well as the oxidation states of the platinum. The study of the Zr 3d core level spectra showed a value of 182.4–182.6 eV of binding energy in all of the catalysts with Zr, which confirms the presence of zirconium as ZrO_2_ species.^50^ The surface concentration of Zr in the catalyst was similar for all three catalysts, although slightly lower in the monometallic catalysts than in the bimetallic ones.

**Table 2:** XPS Data of Zr- or Pt–Sepiolite-Containing Catalysts

		atomic concentrations (%)/binding energy (eV)								
	C 1s		O 1s		Si 2p	Mg 2p	Al 2p	Zr 3d	Pt 4f	
sample	C_avd_	CO_3_^2–^	O^2–^	H_2_O	SiO_4_^4–^	M^2+^	Al^3+^	ZrO_2_	Pt^0^	Pt^2+^
10Zr/Sep	14.19 (284.8)	1.73 (287.5)	55.43 (532.0)	1.10 (534.4)	16.83 (102.7)	8.80 (50.4)	0.04	1.88 (182.4)		
1Pt/Sep	11.75 (284.8)	1.18 (287.5)	55.75 (532.1)	1.20 (534.5)	18.61 (103.0)	11.35 (50.6)	0.03 (74.4)		0.10 (71.6)	0.02 (72.7)
10Zr/1Pt/Sep	15.04 (284.8)	1.08 (287.6)	54.50 (532.0)	1.11 (534.3)	16.21 (102.8)	9.68 (50.4)	0.03 (74.6)	2.18 (182.6)	0.04 (71.4)	0.05 (72.6)
1Pt/10Zr/Sep	14.50 (284.8)	1.07 (287.6)	54.72 (532.0)	1.22 (534.4)	16.86 (102.8)	9.39 (50.4)	0.03 (74.5)	2.04 (182.5)	0.08 (71.6)	0.05 (72.6)

The study of Pt 4f core level spectra showed similar concentrations in all platinum-containing catalysts (0.09–0.13). Two different platinum oxidation states were detected, whose proportion depends on the nature of the catalyst: a binding energy between 71.4 and 71.6 eV for Pt^0^ and 72.6–72.7 eV for Pt^2+^ surface species. In the monometallic Pt catalyst (1Pt/Sep), the atomic concentration of metallic Pt^0^ was much higher than that of the oxidized Pt^2+^ form (Pt^0^/Pt^2+^ = 5). However, in the bimetallic catalysts, platinum was more oxidized, with the Pt^0^/Pt^2+^ ratio being 0.8 and 1.6 for 10Zr/1Pt/Sep and 1Pt/10Zr/Sep, respectively.

**Figure 3:**
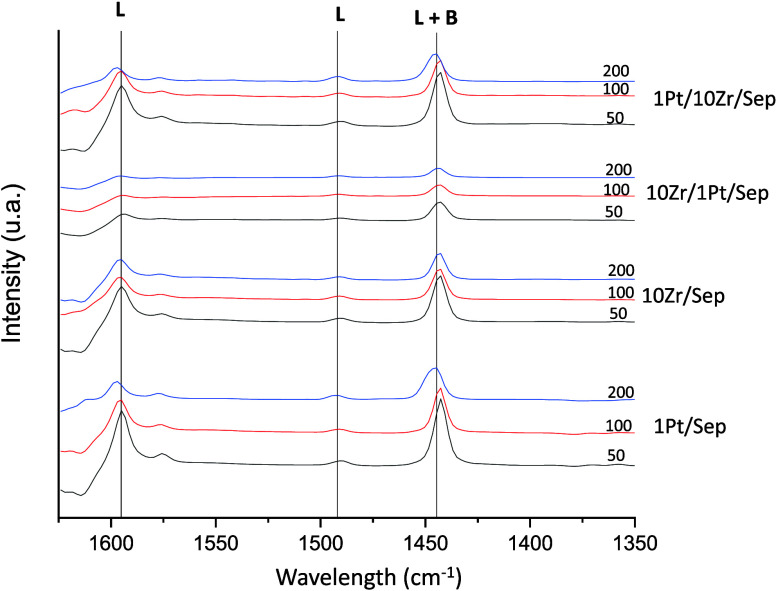
FTIR spectra of adsorbed pyridine at different temperatures for different catalysts.

The acidity properties of catalysts were measured by FTIR-coupled adsorption–desorption of pyridine to determine the amount, nature, and strength of the catalysts’ surface acidity (Figure 3). Vibrations of pyridine adsorbed onto the material were detected at 1595 cm^–1^, 1576 cm^–1^, 1492, and 1444 cm^–1^. The signals at 1595 and 1444 cm^–1^ are assigned to ν_8a_ and ν_19b_ ν(CCN) vibrations, respectively.^51^ Their appearance is related to the coordination of pyridine with strong Lewis acid sites (LASs).^52^ The vibration at 1576 cm^–1^ (ν_8b_) is the result of the interaction of pyridine with weak Lewis acid sites. On the other hand, the band at 1492 cm^–1^ is nonselective and can be produced by pyridine interacting with both Lewis or Brønsted acid sites (BASs).^53,54^ However, none of the characteristic bands typically ascribed to Brønsted acid sites are present in the spectra.^55^ Thus, it can be inferred that the materials display only Lewis acidity or very low Bronsted acidity. As expected, the intensity of the bands decreased after consecutive desorption steps. It is noteworthy that a large fraction of the bands resisted desorption even at 200 °C, denoting a strong interaction between pyridine moieties and active centers on the surface of the material. As it was reported in previous studies,^56^ the absence or very low concentration of BAS was detected when sepiolite was used as a support to synthesize catalysts based on zirconium.

**Figure 4:**
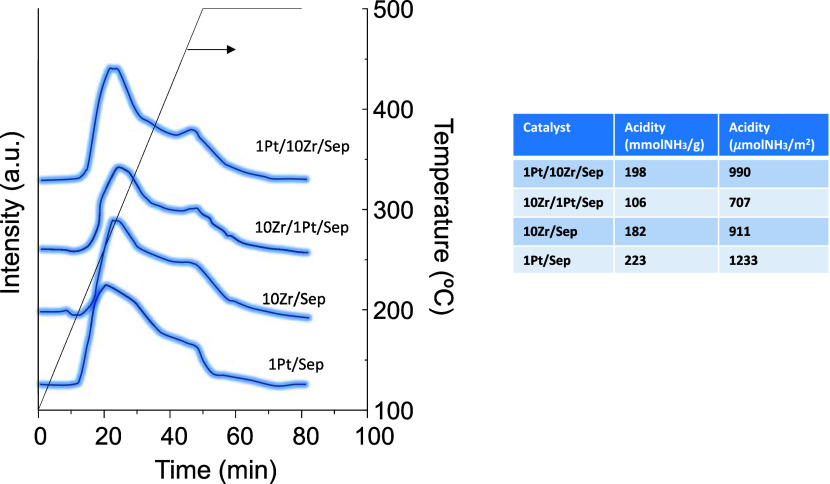
NH_3_-TPD profiles of the different Pt/and/or Zr/Sep catalysts.

The total acidity of these catalysts was also estimated by the temperature-programmed desorption of NH_3_. As shown in Figure 4, the catalyst with the highest acidity per mass of the sample was 1Pt/Sep, followed by 1Pt/10Zr/Sep, 10Zr/Sep, and 10Zr/1Pt/Sep. If the acidity is considered per surface area, the same trend is observed, although it is true that the least acid catalyst was that with the lowest surface area. Part of the acid sites has been reported to proceed from the sepiolite support,^56^ although the addition of Zr leads to an expected increase in the amount of acid sites. Similarly, the addition of Pt led to higher levels of acidity. In all cases, the catalysts display similar profiles, showing the presence of weak and medium acid sites, and also a certain proportion of strong sites.

**Figure 5:**
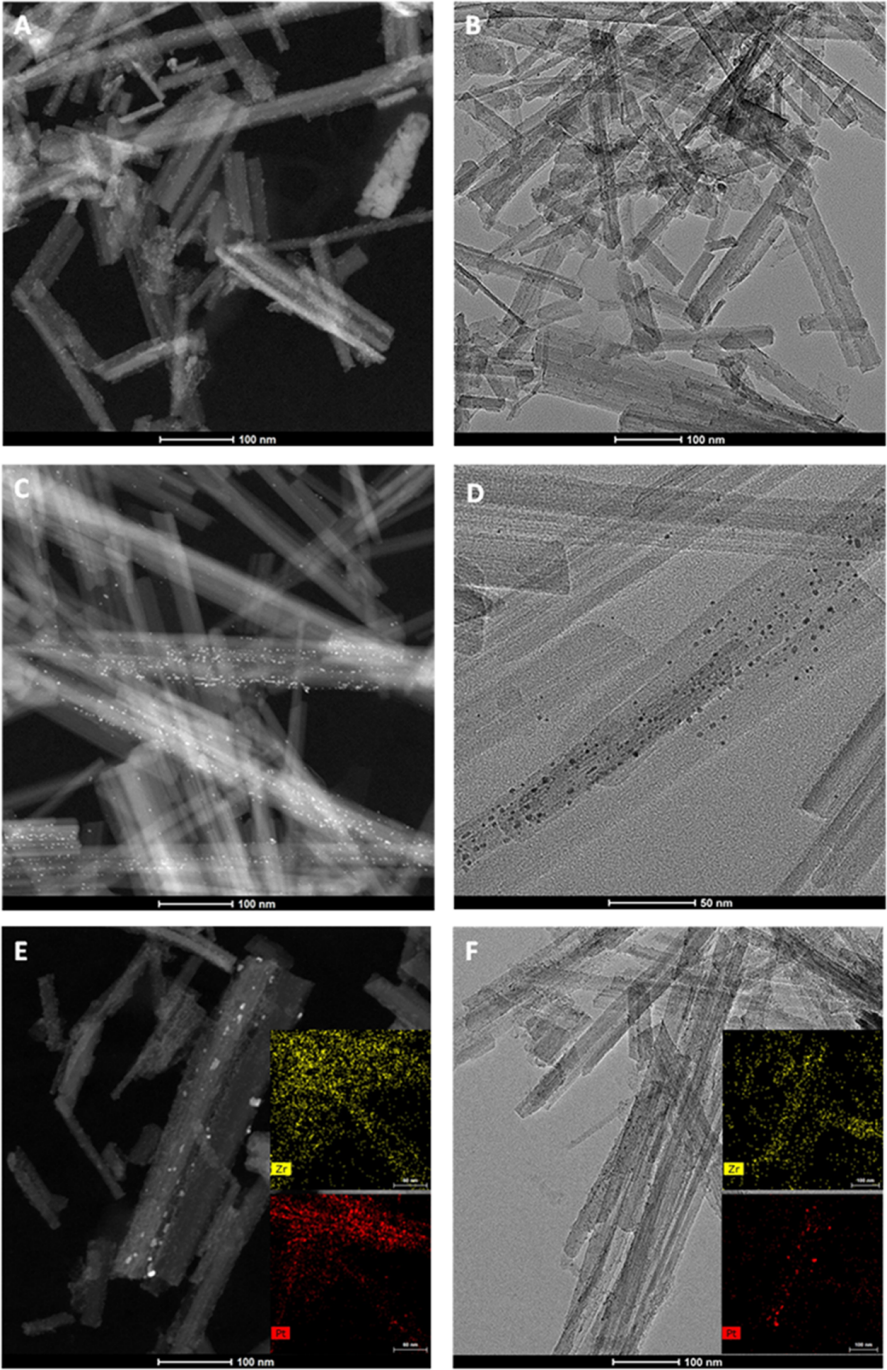
TEM and HAADFSTEM with EDX mapping images of 10Zr/Sep (A, B), 1Pt/Sep (C, D), 10Zr/1Pt/Sep (E), and 1Pt/10Zr/Sep (F).

Figure 5 shows the HR-TEM images of the catalysts synthesized. Figure 5a,b shows the HR-TEM images of the 10Zr/Sep catalyst. The ZrO_2_ nanoparticles were properly dispersed on the surface of the sepiolite fibers (where the nanoparticles covered a great part of the fibers). Images from 1Pt/Sep can be observed in Figure 5c,d. In these figures, platinum nanoparticles were detected on the surface of the sepiolite fibers. The size of the Pt nanoparticles is very low with diameters below 4 nm, most of them in the 1–2 nm range. Figure 5e,f shows the images of the bimetallic catalysts 10Zr/1Pt/Sep and 1Pt/10Zr/Sep, respectively. Although the overall aspect is similar, some differences could be appreciated. Then, depending on the last added metal to the support, part of the active sites of the first metal added could have been blocked under the second metal addition. EDX mapping shows for the 10Zr/1Pt/Sep catalyst that both Zr and Pt are rather homogeneously dispersed on the sepiolite fibers, although there are some zones with a higher concentration of Pt. In the case of the 1Pt/10Zr/Sep catalyst, it is observed that zirconium is well dispersed on the support and, on this zirconium oxide layer, platinum nanoparticles are deposited. In this case, platinum is not so homogeneously dispersed but is present as tiny nanoparticles.

### 3.2 Batch Catalytic Results

**Figure 6:**
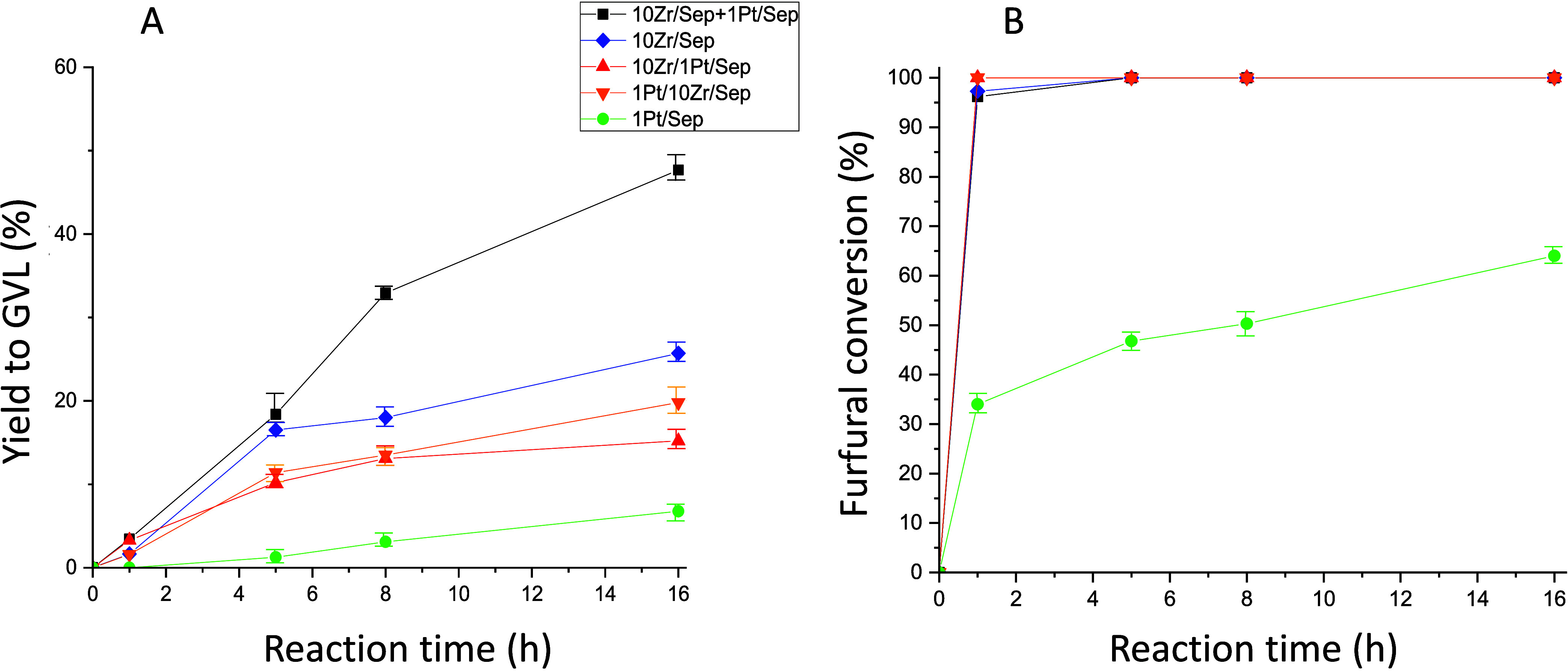
Yields to GVL (A) and FF conversion (B) using different catalysts based on Pt and Zr supported on sepiolite. Reaction conditions: 5 mL of 2-propanol, 0.25 mmol of FF, 0.1 g of catalyst (0.05 g + 0.05 g in the case of the mixture of both catalysts), 180 °C.

The characterized catalysts in the previous section were tested in the transformation of FF in GVL in one pot at 180 °C as a function of reaction time and using 0.1 g of catalyst in each study. A physical mixture of monometallic catalysts (1Pt/Sep +10Zr/Sep) was also tested for this reaction. The yields to GVL using the different catalysts as well as the mixture are shown in Figure 6. The catalyst containing only platinum presented a low yield to GVL even after 24 h of reaction, whereas that with only zirconium showed a moderate yield to GVL, which significantly increases with the reaction time (up to ca. 17% after 8 h). Regardless of the order of addition, the bimetallic catalysts (1Pt/10Zr/Sep and 10Zr/1Pt/Sep) produced similar yields to GVL in all reaction times tested, without exceeding 15% of yield to GVL after 8 h of reaction. Interestingly, the physical mixture of both monometallic catalysts, 1Pt/Sep and 10Zr/Sep (0.05 g of each one), meant a remarkable increase in the GVL formation in comparison to the rest of the catalysts, obtaining a GVL yield of 33% after 8 h of reaction. For the experiment of the physical mixture, the amount of Zr and/or Pt is approximately half of that of the other catalysts. Then, we undertook new experiments with a physical mixture but used 0.1 g of each one. Interestingly, after 8 h of reaction, an enhanced yield of 45% to GVL was obtained. Finally, we want to mention that using the 0.05/0.05 physical mixture, a yield to GVL of 48% was reached by increasing the reaction time to 16 h.

**Figure 7:**
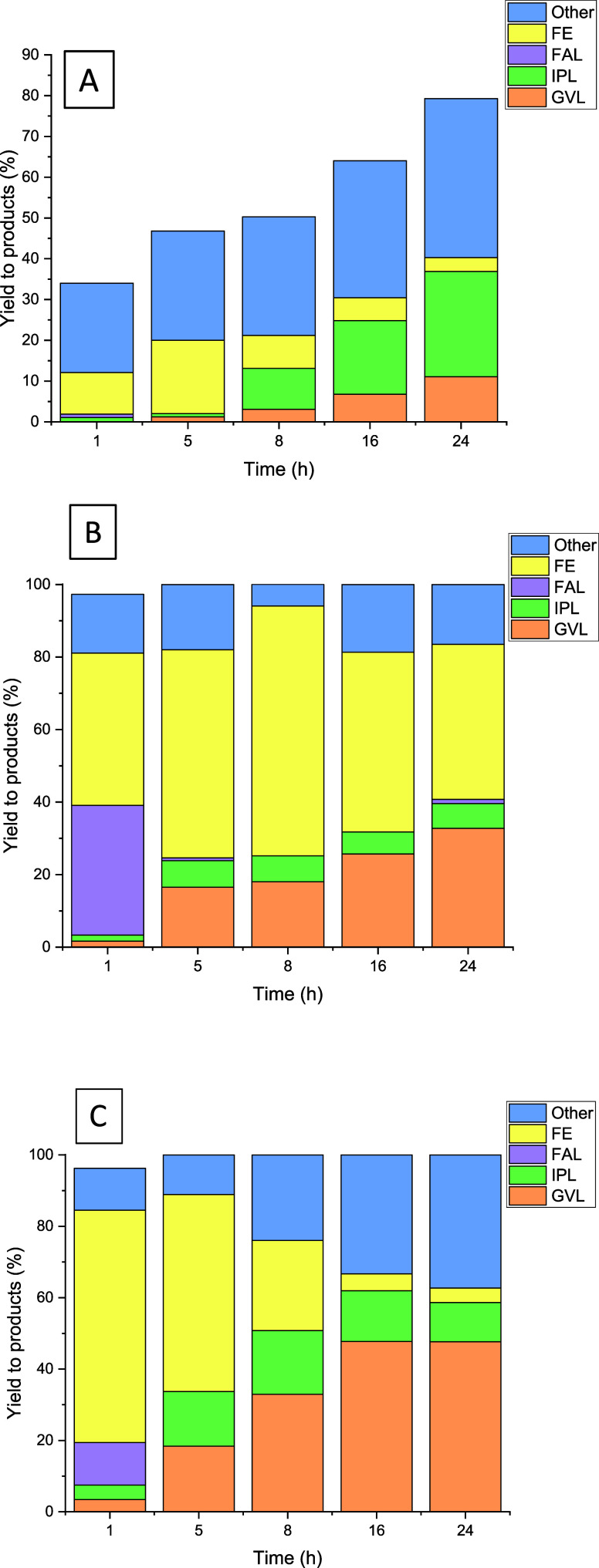
Yields to products in the transformation of FF to GVL in one pot using different catalysts: (A) 10Zr/Sep, (B) 1Pt/Sep, and (C) 10Zr/Sep+1Pt/Sep. Reaction conditions: 5 mL of 2-propanol, 0.25 mmol of FF, 0.1 g of catalyst (0.05 g + 0.05 g in the case of the mixture of both catalysts), 180 °C.

Figure 7 shows the evolution of the yields to the different reaction products with the reaction time for the monometallic catalysts (10Zr/Sep, 1Pt/Sep) and the physical mixture (10Zr/Sep+1Pt/Sep). It can be observed that the 1Pt/Sep catalyst presents lower activity than those containing zirconium. In fact, after 16 h, the FF conversion did not reach 80%. Moreover, with this catalyst, not only GVL is formed with low yields but also the yield toward byproducts (unwanted products) is high. With this catalyst, a high yield of furfuryl ether (FE) was obtained in short reaction times but decreased due to its transformation into IPL. It is noteworthy to mention that after 16 h of reaction, only 6% yield of GVL was produced.

In the case of the catalyst with only zirconium (10Zr/Sep catalyst), the reactivity was high, achieving almost total conversions after just 1 h of reaction. High yields of FAL and FE were initially observed. Moreover, the yield to FE increased along the reaction time, until reaching a maximum of 69% after 8 h of reaction. For longer reaction times, the formation of FE decreased to the detriment of GVL. A maximum of 30% of GVL yield was obtained after 24 h. In the case of IPL, low yields were obtained, regardless of the reaction time. Then, it seems that using the Zr-containing catalysts, the conversion of FE into IPL could be the limiting step in the transformation of FF into GVL, as it was reported in other previous studies.^56^

Using the physical mixture of the monometallic catalysts (10Zr/Sep +1Pt/Sep), the FE yield was high at short reaction times, but the maximum value was obtained at 1 h of reaction, reaching 65%. After this time, the yield decreased in favor of IPL and GVL. This mixture of both catalysts favored the production of GVL, reducing the yields of FE and IPL. A maximum GVL yield of 48% was achieved after 16 h of reaction.

As it was demonstrated in previous studies carried out with sepiolite as a support, the presence of BAS was hardly detected even though GVL was formed.^56^ It indicates that a high proportion of BAS, although desirable, could not be strictly necessary to carry out the transformation of FF in GVL in one pot. Thus, the basicity of the sepiolite support could be beneficial to produce GVL in higher yields, avoiding the production of byproducts, as reported elsewhere.^56−58^

**Figure 8:**
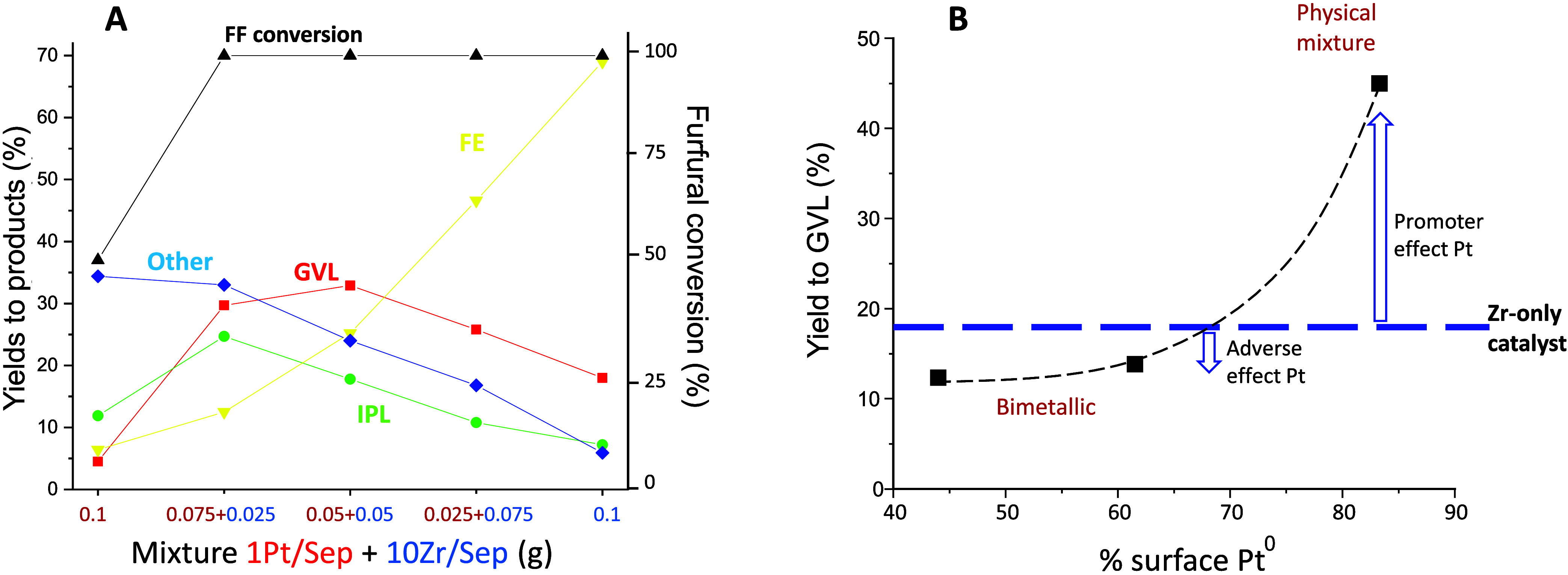
(A) Furfural conversion and yield to products in the transformation of FF into GVL in one pot, varying the amount of each catalyst 10Zr/Sep and 1Pt/Sep. Reaction conditions: 5 mL of 2-propanol, 0.25 mmol of FF, 8 h at 180 °C. (B) Influence of the surface platinum oxidation state on the yield to GVL. Reaction conditions: 5 mL of 2-propanol, 0.25 mmol of FF, 8 h at 180 °C. 0.1 g of bimetallic catalysts. Note: In (b), for the physical mixture, 0.1 g of each sample (10Zr/Sep and 1Pt/Sep) was used in order to have similar Zr and Pt contents than in bimetallic catalysts.

In the present paper, we have shown that the most effective catalytic system to produce GVL in high yields from FF involved a physical mixture of two catalysts based on Pt and Zr supported on sepiolite. Aiming to study the effect of each metal catalyst on the transformation of FF into GVL, mixtures with varying amounts of 10Zr/Sep and 1Pt/Sep were tested. Figure 8a shows the results of the reactions undertaken in all cases at 180 °C for 8 h. When the catalyst 1Pt/Sep was used, only 57.2% of FF was converted, achieving low yields to IPL, GVL, and FE of 11.9, 4.5, and 6.4, respectively. Interestingly, 1Pt/Sep mainly produced other products with selectivities of over 60%. Some of these compounds (analyzed by CG-MS) were either intermediate products, such as α-angelica lactone, or, mainly, byproducts, such as 2-(2-furanylmethyl)–5-methyl-furan or 2,3-(oxybismethylene)bis-furan. Using the catalyst with only Zr (10Zr/Sep), a high yield to FE of 69% was observed, whereas the formation of GVL was only moderate (yield 18% under these reaction conditions).

Interestingly, the use of any physical mixture (regardless of the proportion) showed an increase in the GVL formation compared with monometallic catalysts. Then, a clear trend was observed when the composition of the mixture was varied. Thus, when the proportion of the platinum catalyst increased, the formation of other products increased, whereas the yield to FE decreased. In the case of GVL formation, there was an optimal mixture that corresponds to a mass ratio of 1:1 (0.05 g of each catalyst).

The improved performance of the physical mixture contrasts with the results observed in bimetallic PtZr catalysts, which behave even worse than the monometallic Zr catalyst. According to the characterization results, the bimetallic catalysts present a higher mean oxidation state of platinum than the monometallic Pt catalyst. Therefore, the promoter effect and optimal behavior of the physical mixture may be related to the main presence of metallic platinum. Figure 8b plots the GVL formation with the surface oxidation state of platinum. It is observed that the option (physical mixture) in which platinum is predominantly present as Pt^0^ leads to the highest yield to GVL. As the amount and oxidation state of surface Zr as well as its dispersion on the sepiolite support are very similar for monometallic Zr and bimetallic catalysts, the different catalytic results obtained must be related to the characteristics of the platinum sites.

In the present article, we have observed the promoter effect of platinum even though the catalyst containing platinum alone does not favor the formation of GVL. The promoter effect of platinum could be related to the high capacity of platinum sites for carrying out the ring-opening (FE into IPL) since the use of the platinum catalyst results in an increase in the IPL yield at the expense of FE. On the other hand, the presence of zirconium sites favors both the hydrogenation and cyclization of FF toward IPL into GVL. Therefore, the simultaneous presence of platinum and zirconium favors the overall cascade reaction.

There are two trends. By increasing the amount of the Pt sites, the formation to other products is favored, whereas the IPL to GVL step is hindered. However, by increasing the amount of Zr, the transformation of FE to IPL is not favored. Then, there is a trade-off in which among the mixtures tested, the 1:1 ratio is the optimal one.

**Scheme 1:**
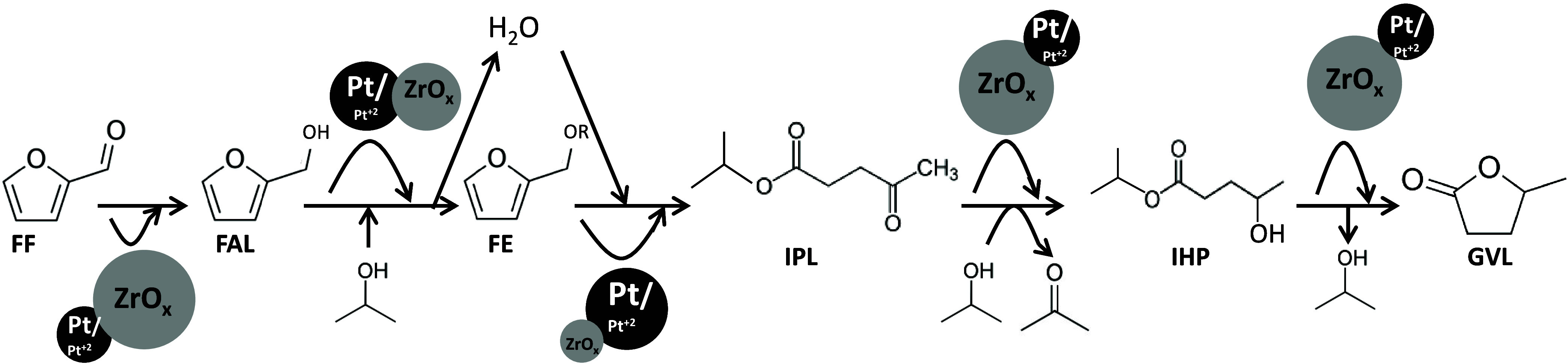
Reaction Mechanism of Production of GVL from FF and the Role of Pt and ZrO_*x*_

If all of the experiments varying the amounts of each catalyst added into the reaction media are considered, specific roles of each metal in the cascade reaction of conversion of FF into GVL in one pot could be proposed (Scheme 1). In the first step, the hydrogenation of FF into FAL, ZrO_*x*_ sites play the most important role, although Pt sites can activate FF but with lower reactivity. In fact, the catalyst containing only Zr was able to convert all the FF after only 1 h, whereas the catalyst with only Pt hardly could convert 34% of FF. On the other hand, the catalyst with only Pt or the mixture with more Pt presents high selectivity to IPL, much higher than those with Zr or mixtures with more Zr. Then, the conversion of FE into IPL is likely favored by the platinum sites. The further transformation of IPL to GVL is mainly carried out by the zirconium sites. However, in the case of using only a zirconium oxide catalyst, a high yield of FE was detected, suggesting a slower transformation of FE into IPL.

Recycling tests of the mixture of Pt and Zr catalysts was carried out in order to investigate the stability of this mixture (Figure S4). A catalyst was tested to an 8 h reaction and recycled three times. It was found that the yield to GVL hardly varied after three cycles, although some variations in the formation of IPL, FE, and other products were found.

### 3.3 Continuous Catalytic Results

Due to the good results obtained using batch reactors, we have decided to test the whole set of catalysts (mono- and bimetallic as well as the physical mixture) in continuous flow conditions, employing a homemade liquid-phase fixed-bed reactor (described in paragraph 2.5). Thus, we wanted to check if these catalysts, especially the physical mixture, are also efficient when working with a continuous reactor.

**Figure 9:**
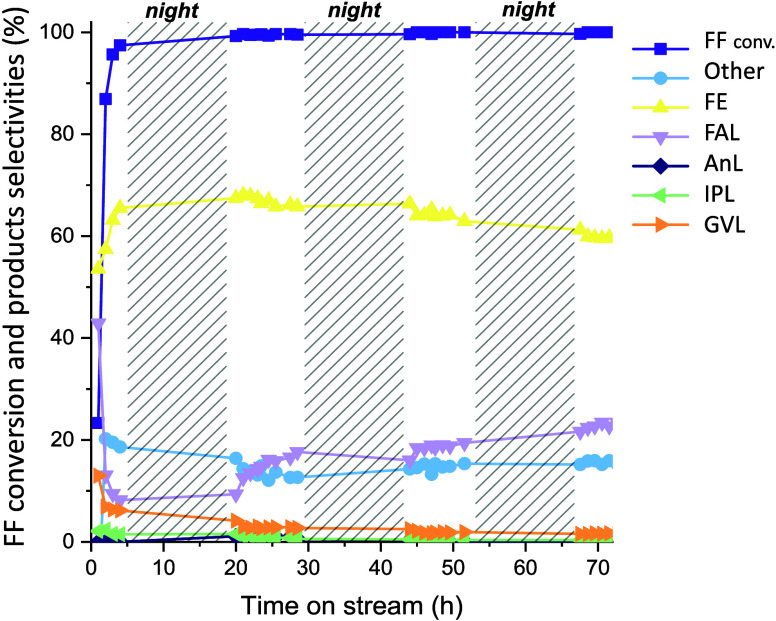
FF conversion and product selectivities as a function of time(h) on 1Pt/10Zr/Sep. Reaction conditions: [FF] = 67 mM, τ = 10 min, *T* = 180 °C, *m*_cat_ = 0.5 g.

First of all, the long-term stability of the materials was investigated. The catalytic performance of 1Pt/10Zr/Sep (Figure 9) is reported as an example. Figure 9 shows the trend of furfural conversion and selectivity to the main products as a function of reaction time on stream. The total conversion of furfural was observed in the conditions tested. Except for the first 3 h of the reaction, to reach the stationary state in the reactor, the catalyst demonstrated to be very stable in the reaction conditions chosen, maintaining activity for 4 consecutive days. Indeed, although the selectivity to FAL increased during the test performed for 4 days, the selectivity to the major product, FE, decreased only 5% in 72 h. Moreover, XRD analysis of the catalyst before and after the reaction (Figure S5) did not show any structural change of the catalyst; therefore, these results are in good agreement with the relatively stable catalytic activity.

**Figure 10:**
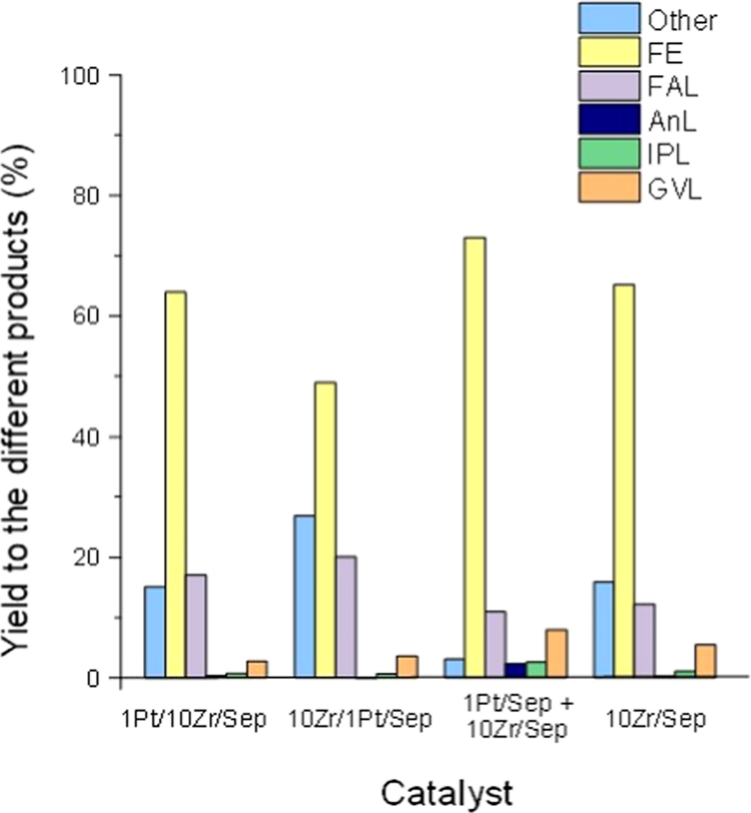
Catalyst performance comparison for the one-pot reaction from FF to GVL. Reaction conditions: [FF] = 67 mM, τ = 10 min, *T* = 180 °C, *m*_cat_ = 0.5 g.

After proving the long-term stability of all catalysts (Figure S6), the mean catalytic results of every test (mediated on 7 h time on stream) were compared as shown in Figure 10. Total conversion of furfural was always observed in all catalysts or a combination of catalysts. The major product was FE in all cases with selectivity between 50 and 70%. Furthermore, the production of nonintermediate to GVL products remained under 20% except in the case of the 10Zr/1Pt/Sep catalyst. Unfortunately, the GVL selectivity did not exceed more than 8% in any of the experiments. Interestingly, the physical mixture of monometallic catalysts led to the highest space yield time (STY) for both GVL and FE (Table S1), with the lowest formation of other products, which are not intermediates of GVL.

The reason for the absence of 1Pt/Sep in Figure 10 is the formation of a plug in the reactor that prevented the solution from flowing through it when this catalyst was tested. Different parameters were changed trying to solve the problem like using bigger pellets or pressing the pellets for longer times. However, the results did not change. In any case, the worst catalytic results in terms of both catalytic activity and formation of GVL were expected with the monometallic Pt catalyst.

### 3.4 Comparison between Batch and Continuous Flow Conditions

**Figure 11:**
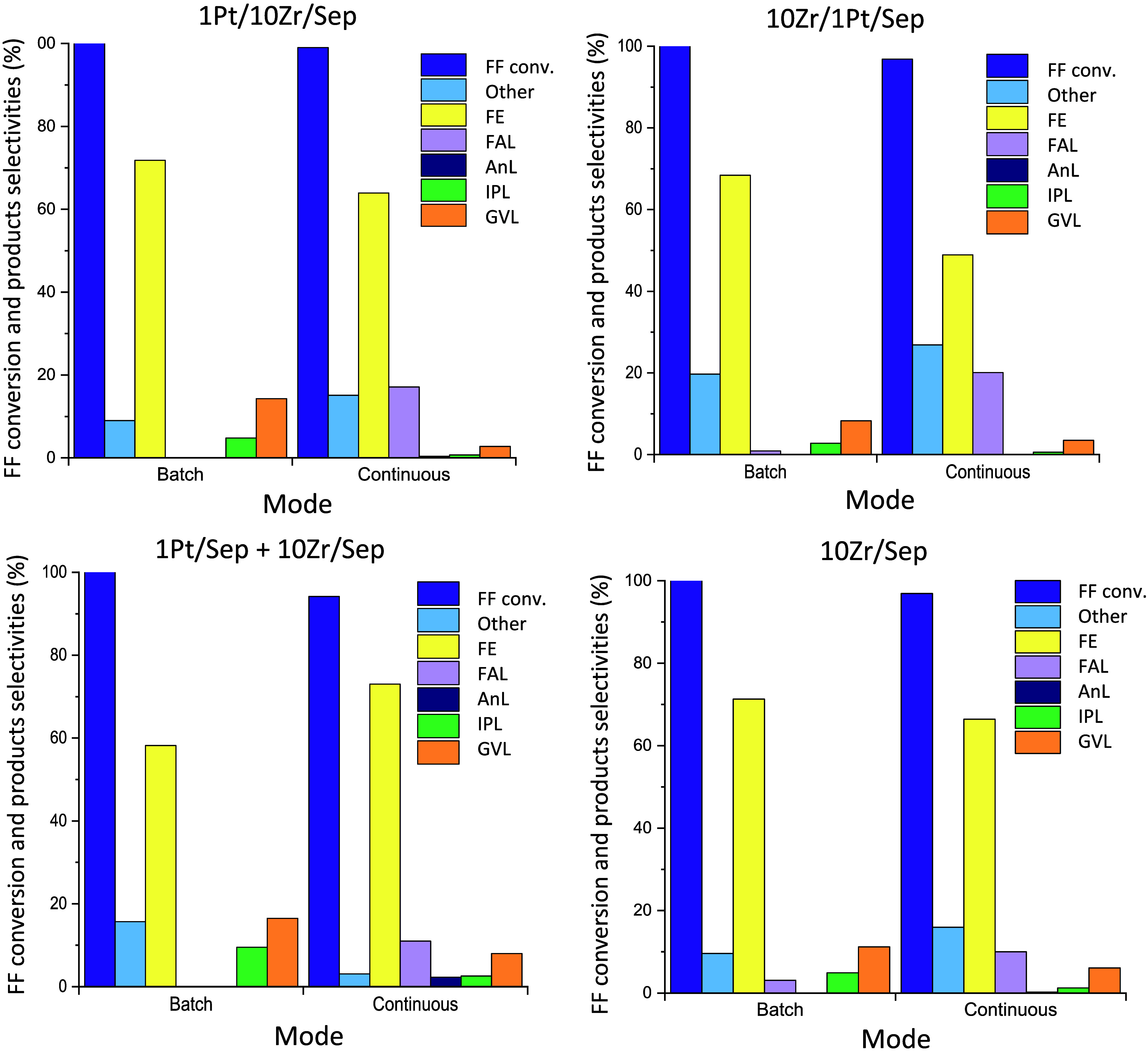
Comparison between batch and continuous catalytic performances of the synthesized materials in the one-pot reaction from furfural to GVL. Batch reaction conditions: 5 h, 180 °C, 5 mL of 2-propanol, 0.25 mmol of FF, and 0.1 g of catalyst. Continuous reaction conditions: [FF] = 67 mM, τ = 10 min, *T* = 180 °C, and *m*_cat_ = 0.5 g.

It has been demonstrated that the batch-to-continuous transition for the production of large-volume specialty chemicals yields strong process intensification benefits. Indeed, performing under flow conditions can provide shorter reaction time, fast reagent mixing, better heat transfer, simpler downstream processing, easier scale-up, and increased reactor volume productivity. This is the reason why in the present work the catalytic performances of Pt and Zr catalysts in converting furfural to GVL were tested in continuous flow, as well. Consequently, the results obtained were compared with the results previously obtained working in batch mode. Figure 11 shows the results by comparing the batch and continuous flow experimental conditions for each catalyst. The conversion of furfural was complete (or almost) and the selectivities to FE were similar in each case. However, working in continuous mode lowered selectivity toward GVL, and better selectivities toward FAL were obtained. Furthermore, the formation of other products seems to be favored in continuous mode, with the values being higher than the ones obtained in batch mode. An encouraging result was observed in the case of 1Pt/Sep +10Zr/Sep, in which this value was nearly zero.

Overall, the comparison of the catalytic data between the batch and continuous modes shows a good agreement with respect to the overall trend, as the physical mixture showed the highest GVL formation and the lowest formation of other products. However, the yields to GVL were remarkably higher under batch conditions. These different results could be due to a higher sensitivity to the presence of Brønsted acid sites when working in a continuous regime. The high selectivity to FE, contemporarily to the low selectivity to GVL, suggests a major lack of Brønsted acidity in all of the materials. This hypothesis is confirmed by the FTIR-pyr performed. Indeed, FE needs the presence of Brønsted acid sites to form IPL, and consequently GVL. On the other hand, given that the total conversion of FF was reached and hardly any IPL or AnL were detected, Lewis acidity seems to be enough to catalyze the steps that need it, hydrogenation, estherification, and cyclation. Alternatively, it could be also possible that, as the physical mixture presents the lowest formation of other products, being the selectivity to GVL and its intermediates very high, experiments in continuous regime with lower space velocity could favor the advance of the overall reaction promoting the transformation of the intermediates and then increasing the yield to GVL.

## 4 Conclusions

Pt and/or Zr catalysts have been characterized by several techniques, including XRD, TEM, FTIR of adsorbed pyridine, N_2_ adsorption, and NH_3_-TPD, and tested in the transformation of furfural (FF) into γ-valerolactone (GVL). While Pt catalysts present low catalytic activity, any Zr-containing catalyst (or mixture) presents high catalytic activity, easily achieving 100% conversion at low reaction times. One interesting aspect of this work is the fact that a physical mixture of two catalysts based on Pt and Zr led to a significant production of GVL in the one-pot transformation of FF in a batch reactor. In fact, the GVL yield obtained was higher than that of an equivalent catalyst containing only Zr, although the catalyst containing only Pt presented a poor performance. The synergetic effect observed between Pt and Zr is likely because each metal plays an important role in different reaction steps of the conversion of FF into GVL. Thus, the Lewis acid sites related to the Zr sites activate furfural in an easier way and are highly efficient in the hydrogenation/cyclization of IPL into GVL, whereas the presence of Pt sites seems to favor the FE to IPL step. In order to get this promotional effect, it is important that platinum be in a metallic state (such as in the physical mixture) since a deleterious catalytic performance has been observed in bimetallic catalysts containing both Pt and Zr, in which the amount of oxidized platinum is elevated. On the other hand, the comparison of batch and continuous mode demonstrated a good agreement between the two operational methods except for the GVL selectivity, which was always higher working in batch mode. It is noteworthy that there is high production of GVL and its intermediates and low selectivity to undesired products using the physical mixture. For both situations, batch and continuous, the optimal catalyst presents good stability.

## Supporting Information Available

In the Supporting Information file, the scheme of the liquid-phase continuous reactor used is shown (Figure S1). Moreover, the N_2_ adsorption/desorption isotherms as well as the pore distribution have also been included (Figures S2 and S3). Figure S4 shows the results of the reuse tests, whereas Figure S5 presents the XRD patterns of the used catalysts. Figure S6 includes complete information about the tests in continuous regime. Finally, Table S1 presents the space time yield (STY) to FE and GVL on the different catalysts or mixtures (PDF)
