# Supplementary material for: Promoter Effect of Pt on Zr Catalysts to Increase the Conversion of Furfural to γ-Valerolactone Using Batch and Continuous Flow Reactors: Influence of the Way of the Incorporation of the Pt Sites
Source: Energy Fuels. 2024 May 24;38(11):9849–61. doi: 10.1021/acs.energyfuels.4c01174 (PMC11164049; doi:10.1021/acs.energyfuels.4c01174)
Supplement: Supplementary file 1 — ef4c01174_si_001.pdf [file ef4c01174_si_001.pdf]

# Supplementary material

**Promoter effect of Pt on Zr catalysts to increase the conversion of furfural to γ-valerolactone using batch and continuous flow reactors: influence of the way of the incorporation of the Pt-sites**

Adrian García,[a] Anna Saotta,[b] Pablo J. Miguel,[a] Rita Sánchez-Tovar,[a] Giuseppe Fornasari,[b] Alessandro Allegri,[b] Benjamín Torres-Olea,[c] Juan Antonio Cecilia,[c] Stefania Albonetti,[b,*] Nikolaos Dimitratos,[b,*] Benjamin Solsona.[a,*]

[a] Dept. Chemical Engineering, Universitat de València. Av. Universitat s/n, 46100 Burjassot, Valencia, Spain. Benjamin Solsona (Benjamin.solsona@uv.es)

[b] Dept. Industrial Chemistry "Toso Montanari", Università di Bologna, Viale Risorgimento 4, Bologna 40136, Italy. Nikolaos Dimitratos (nikolaos.dimitratos@unibo.it), Stefania Albonetti (Stefania.albonetti@unibo.it)

[c] Dept. Inorganic Chemistry, Crystallography and Mineralogy, Campus de Ciencias, Universidad de Málaga, 29071 Málaga, Spain

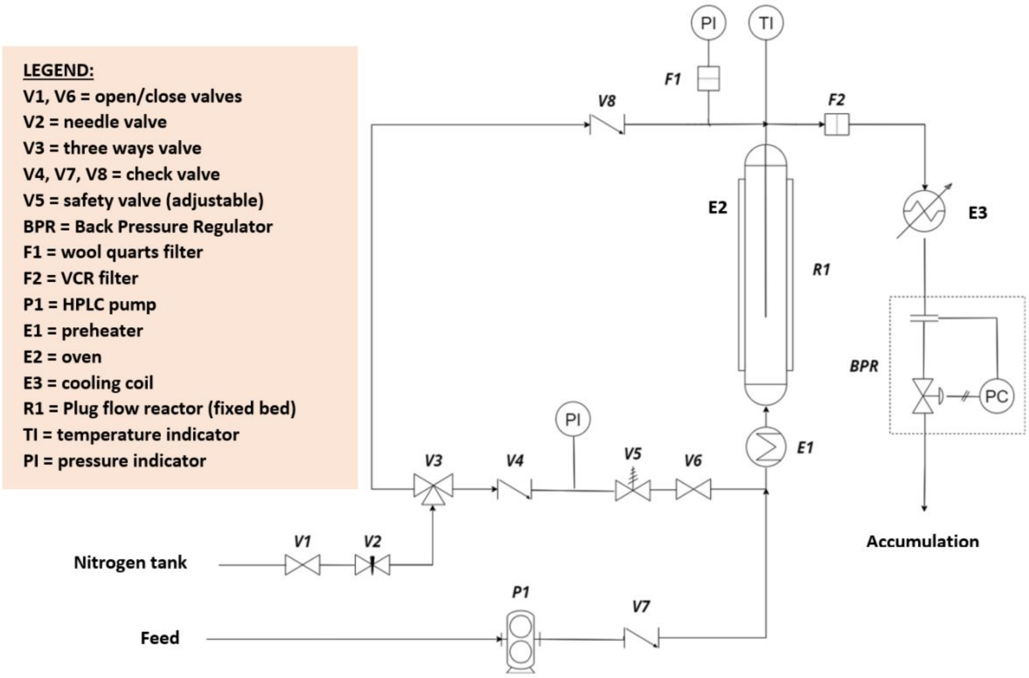


**Figure S1.** Scheme of the liquid-phase continuous reactor used.

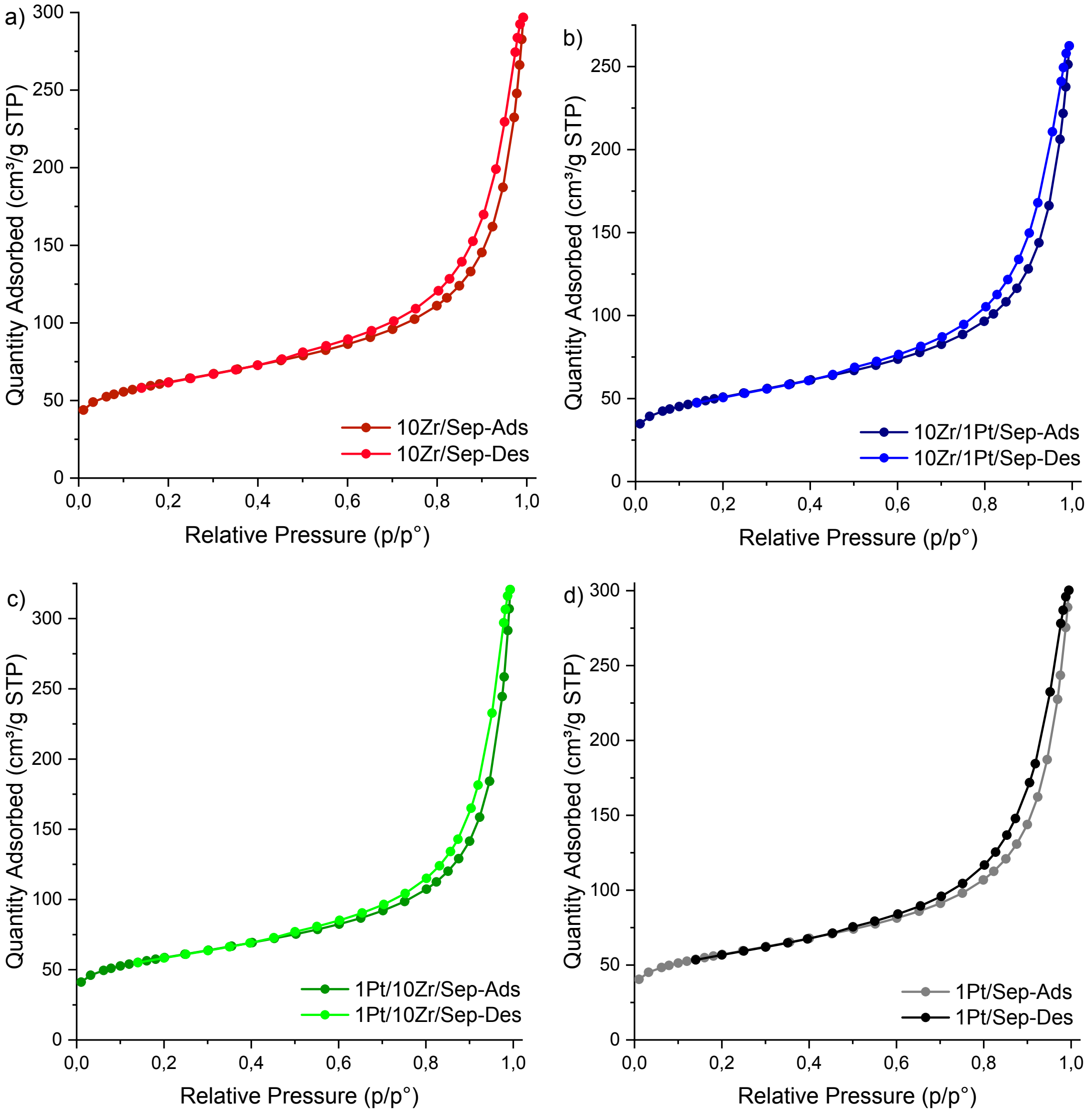


**Figure S2.** $N_2$ adsorption/ desorption isotherms of 10Zr/Sep (a), 10Zr/1Pt/Sep (b), 1Pt/10Zr/Sep (c) and 1Pt/Sep (d).

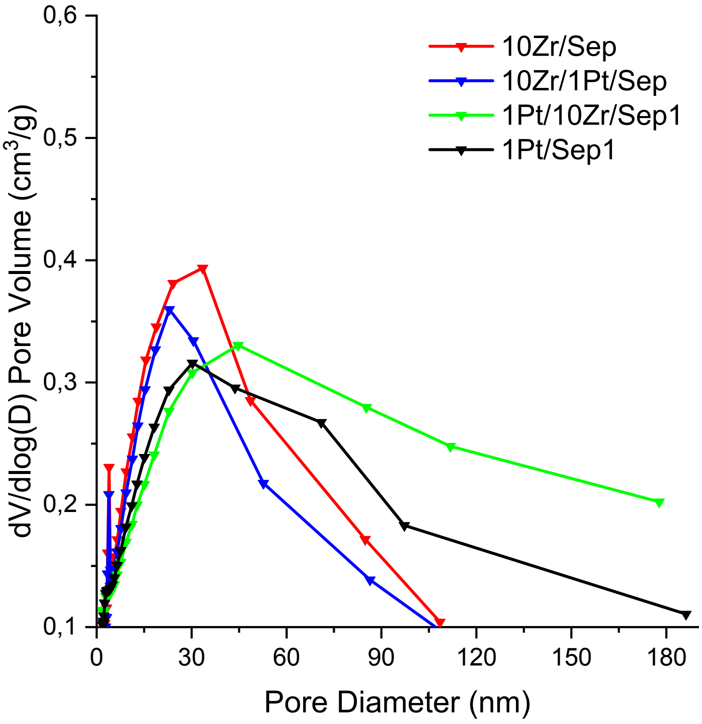


**Figure S3.** Pore distribution curves of 10Zr/Sep (red), 10Zr/1Pt/Sep (blue), 1Pt/10Zr/Sep (green) and 1Pt/Sep (black).

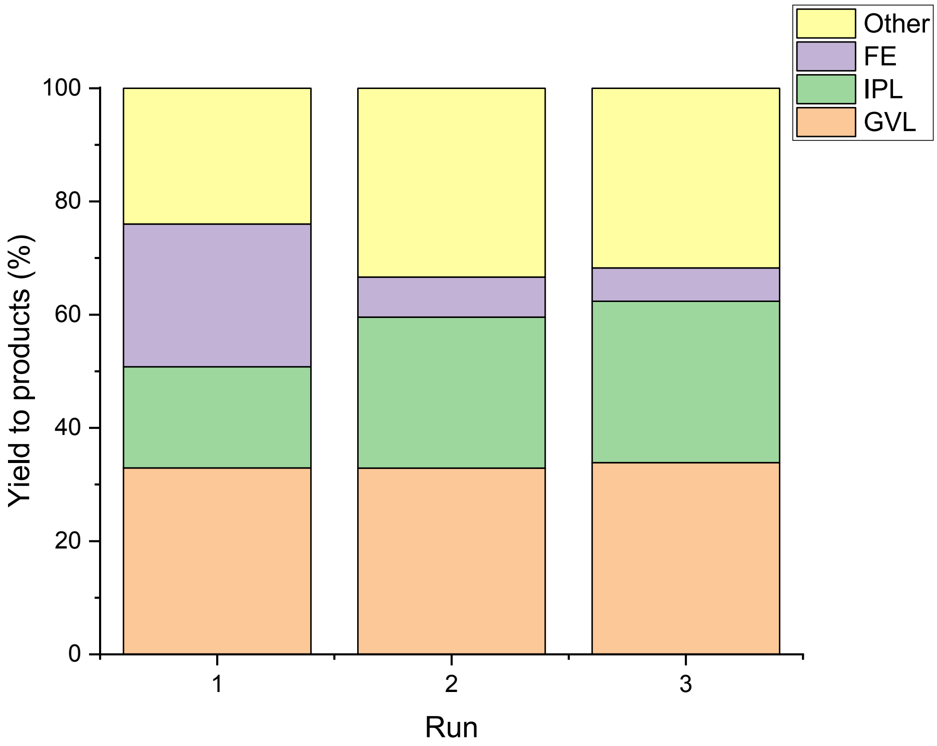


**Figure S41**. Re-use testing of mixture of 1Pt/Sep and 10Zr/Sep to produce GVL from FF in one-pot. Reaction conditions: Batch, 5 mL of 2-propanol, 0.25 mmol of FF, 0.05 g of each catalyst, 8 h at 180 ºC.

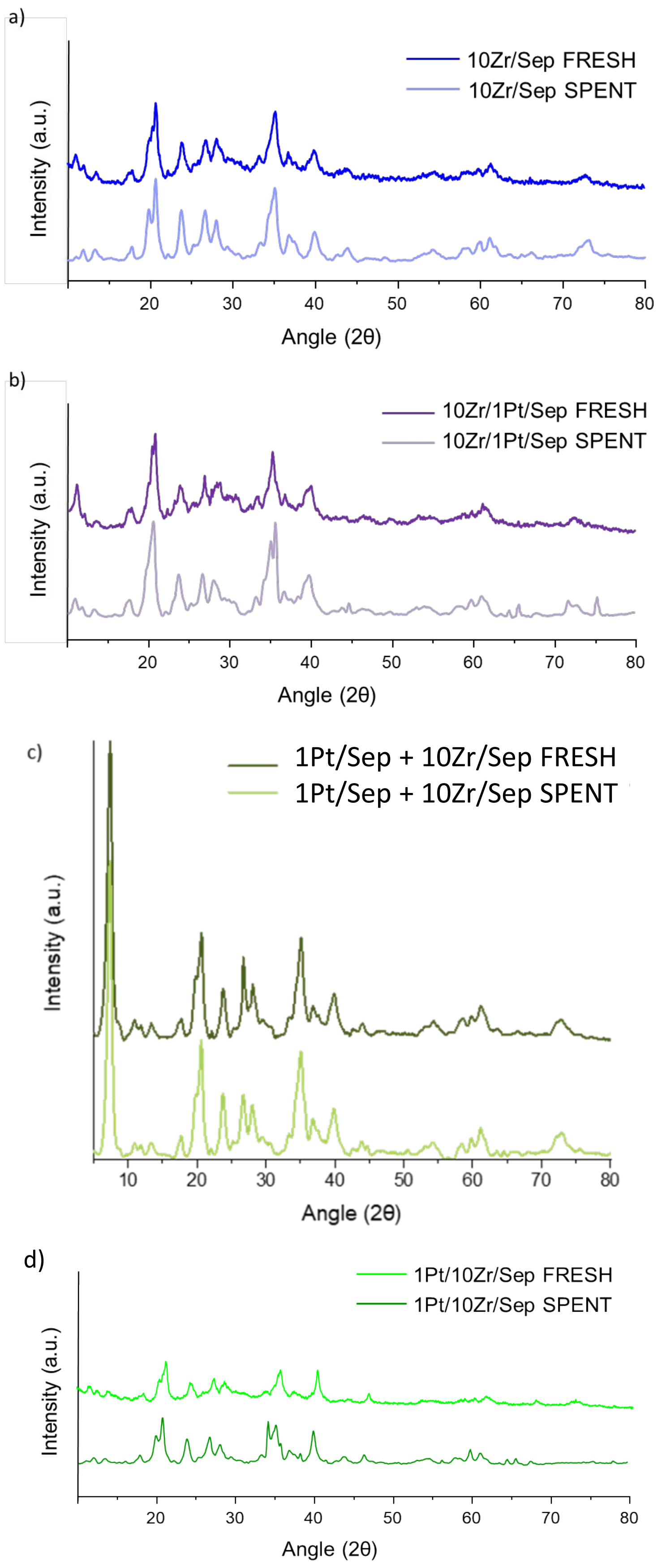


**Figure S5**. XRD patterns of catalyst before (fresh) and after (spent) reaction: (a) 10Zr/Sep, (b) 10Zr/1 Pt/Sep, (c) 1Pt/Sep + 10Zr/Sep and (d) 1Pt/10Zr/Sep.

The graph presented in Figure S6 displays the trend in furfural conversion and selectivity in the products of the cascade reaction from FF to GVL, using all Pt and Zr catalysts introduced in this work (continuous regime) which include 10Zr/Sep, 10Zr/1Pt/Sep, 1Pt/Sep + 10Zr/Sep. Each test was conducted at 180°C, with a contact time of 10 minutes while using a furfural solution with a concentration of 67mM and a mass of catalyst between 0.42/0.52 g.

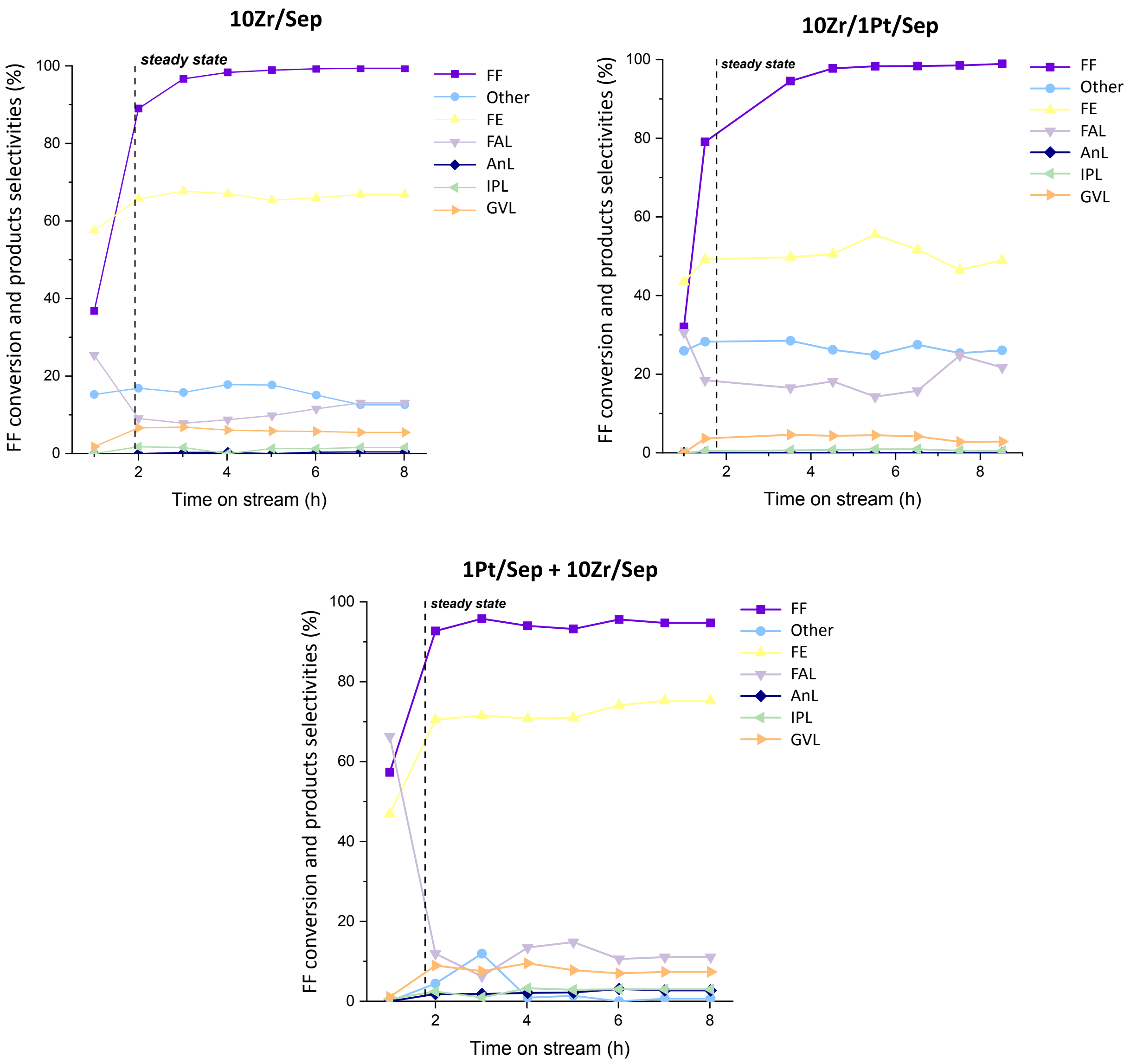


**Figure S6.** Furfural conversion and product selectivities (%) as a function of time (h) over 8h on 10Zr/Sep, 10Zr/1Pt/Sep, 1Pt/Sep + 10Zr/Sep. Reaction conditions: [FU]=67mM, τ=10 min, T=180 °C, $m_{cat}$ between 0.42 and 0.52 g.

**Table S1.** Space time yield (STY) to FE and GVL on the different catalysts or mixtures in continuous experiments. Reaction conditions: [FF] = 67 mM, τ=10 min, T = 180 °C, $m_{cat}$ = 0.5 g, considering steady state at time on stream of 7 h.

| Catalyst | STY FE ($10^{-3}$ $g_{FE}$ $g_{cat}^{-1}$ h) | STY GVL ($10^{-3}$ $g_{GVL}$ $g_{cat}^{-1}$ h) |
|---|---|---|
| 10Zr/Sep | 6.5 | 0.34 |
| 10Zr/1Pt/Sep | 5.0 | 0.23 |
| 1 Pt/10Zr/Sep | 6.5 | 0.19 |
| 1Pt/Sep + 10Zr/Sep | 7.6 | 0.60 |
